# Potential Biomarkers in Systemic Lupus Erythematosus

**DOI:** 10.31662/jmaj.2025-0190

**Published:** 2025-07-07

**Authors:** Yujie Song, Michihito Kono

**Affiliations:** 1 Department of Rheumatology, Endocrinology and Nephrology, Faculty of Medicine and Graduate School of Medicine, Hokkaido University, Sapporo, Japan

**Keywords:** systemic lupus erythematosus, biomarker, immune cell subset, cytokine

## Abstract

Systemic lupus erythematosus (SLE) is a complex autoimmune disorder characterized by heterogeneous clinical manifestations and diverse autoantibody production. Despite advances in treatment, many patients experience disease flares throughout their lives, and current biomarkers like anti-double-stranded DNA antibodies and serum complement levels have limitations in accurately reflecting disease activity. This review examines emerging and established biomarkers for SLE diagnosis, disease activity monitoring, and treatment response prediction.

We discuss immune cell subsets as potential biomarkers, focusing on plasmacytoid dendritic cells, T cell and B cell subsets, especially focused on T cell subsets. The review highlights how imbalances in these cellular populations correlate with disease activity and specific organ involvement. Furthermore, we discuss cytokines, chemokines, autoantibodies, and complement as biomarkers in SLE.

The identification and validation of reliable biomarkers in SLE will ultimately improve clinical decision-making regarding treatment selection, glucocorticoid tapering, and prediction of disease remission, leading to more personalized and effective management strategies.

## Introduction

Systemic lupus erythematosus (SLE) is a complex autoimmune disorder characterized by various autoantibody production and widespread organ inflammation with heterogeneous clinical manifestations ^(1), (2)^. Although immunosuppressants and biologics have improved the prognosis and quality of life of the patients with SLE ^(3), (4)^, many patients experience flares in their life ^(5), (6), (7), (8)^. Some symptoms of SLE are very difficult to diagnose, whether they are due to the disease or other reasons, including infections and drug side effects. Anti-double-stranded DNA (anti-dsDNA) antibodies and serum complement are used as biomarkers of SLE in the clinical setting, but some patients do not have abnormalities in these tests. Identification of reliable biomarkers is requisite for early diagnosis, monitoring disease activity, predicting flares, and evaluating treatment responses. Thus, more useful biomarkers are desired in this field. In this review, we discuss the biomarkers in SLE, including immune cell subsets, cytokines, chemokines, and autoantibodies (Table 1).

**Table 1.** Biomarkers in Systemic Lupus Erythematosus.

Candidate biomarkers	Utility in SLE	Notes
Immune cell subsets		
CD4^+^ T lymphocytes		
T helper 1 cells	disease activity	mucocutaneous involvement and lupus nephritis (LN)
T helper 2 cells	disease activity	produce interferon (IFN)-γ
T helper 17 cells	disease activity	main source of interleukin (IL)-17; LN
Follicular helper T cells	disease activity	debates on which one is authentically flare-correlated
Peripheral helper T cells		
Regulatory helper T cells	disease activity	defective regulation; LN
Age-associated T helper cells	disease activity	
CD8^+^ T lymphocytes	disease activity	
Double-negative T cells	disease activity	important source of IL-17
Plasmacytoid dendritic cells	disease activity	produce type I IFNs
B lymphocytes	disease activity	B cell-targeted therapy and chimeric antigen receptor (CAR) T therapy
Autoantibodies		
Antibodies to double-stranded DNA (anti-dsDNA)	diagnosis	diagnostic criteria
	disease activity	LN
Antibodies to Smith antigen (anti-Sm)	diagnosis	diagnostic criteria; low sensitivity
	disease activity	LN
Antibodies to ribosomal proteins (anti-RibP)	diagnosis	low sensitivity
	disease activity	central nervous system (CNS) lupus
Antibodies to small nuclear ribonucleoprotein (anti-snRNP)	disease activity	CNS lupus
Antiphospholipid antibody (aPL)	disease activity	CNS lupus (?)
Cytokines and chemokines		
BAFF/APRIL system:		
B cell-activating factor (BAFF), A proliferation-inducing ligand (APRIL), B cell maturation antigen (BCMA)	disease activity	cardiovascular involvement and LN
Type I IFNs: IFN-α, IFN-β, IFN-ω	disease activity	LN
Type II IFNs: IFN-γ	disease activity	
Surrogate IFN-regulated markers:		
Interferon γ-inducible protein 10 (IP-10), Galectin 9 (Gal-9), Sialic acid binding Ig-like lectin 1 (SIGLEC1)	diagnosis (SIGLEC1 only)	better for excluding SLE in suspected cases
	disease activity	lack of adequate and convincing evidence
Interleukin-1 families:		
IL-1Receptor antagonist (IL-1Ra), IL-18, IL-36α, IL-36γ, IL-1R2, soluble IL-1R4, Soluble suppression of tumorigenicity 2 protein (sST2)	diagnosis (IL-1Ra only)	
	disease activity	not specific to organ
IL-6	disease activity	not specific to organ
IL-17	disease activity	LN
IL-23	disease activity	LN
Complement system		
C3, C4, CH50	diagnosis	diagnostic criteria
	disease activity	unstable correlation
C3a	disease activity	thrombophilia
C3dg	diagnosis	

## Immune Cell Subsets as Biomarkers in SLE

**Figure 1.**
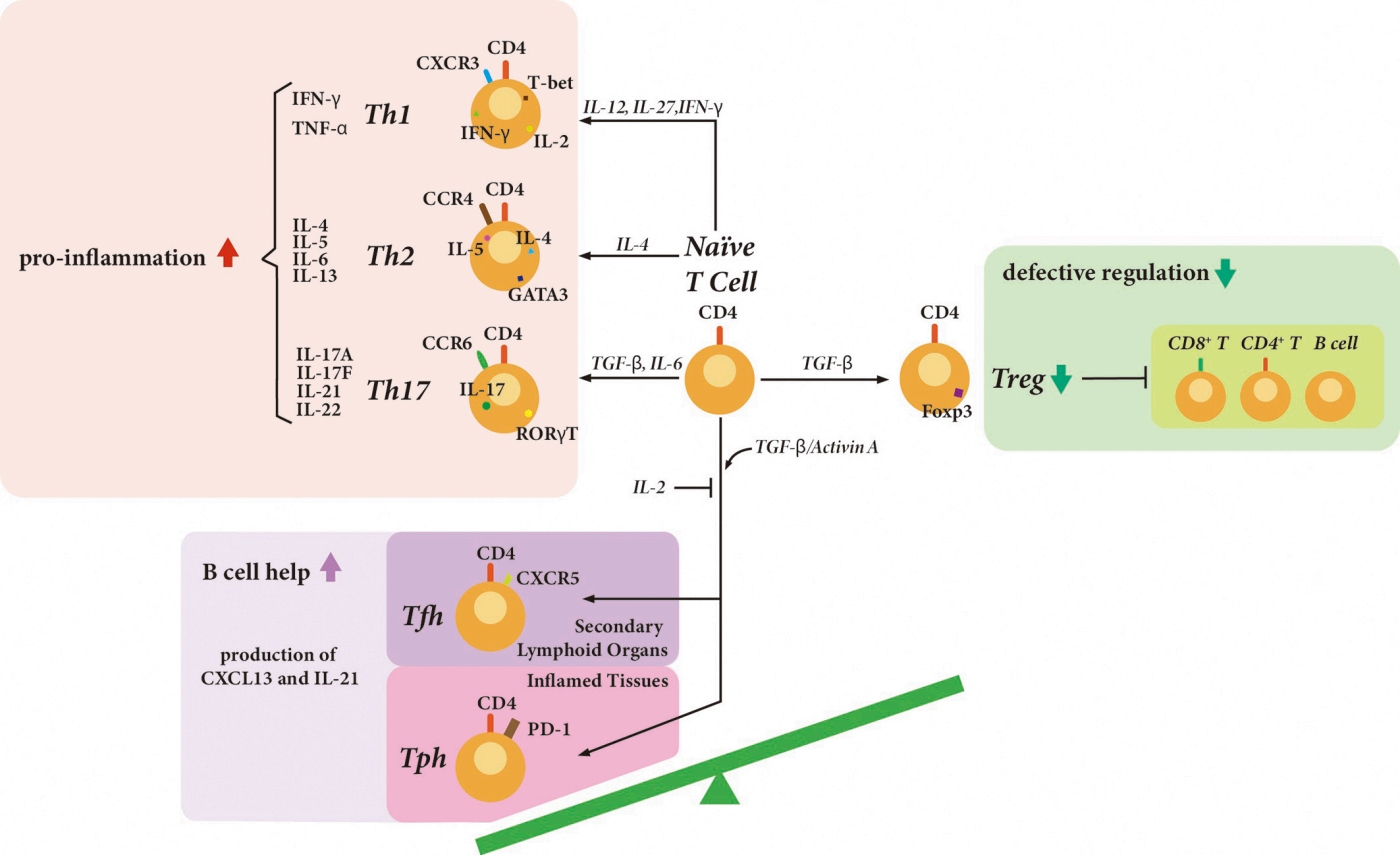
The pathogenesis of systemic lupus erythematosus focusing on T cell and B cell subsets. Naïve T cells differentiate into specific subsets in response to respective cytokine stimulations. In SLE, the differentiation into Th1, Th2, and Th17 subsets is upregulated. The expanded Th1, Th2, and Th17 subsets produce key pro-inflammatory and inflammatory cytokines such as TNF-α, IL-6, IFN-γ, and IL-17. Consequently, patients with SLE are prone to developing inflammation. Moreover, the proportions of Tfh and Tph subsets differentiated from naïve T cells through the stimulation of TGF-β and activin A are increased in secondary lymphoid organs and inflamed tissues, respectively. Their production of CXCL13 and IL-21 helps the proliferation and maturation of B cells, subsequently promoting the production of autoantibodies. At the same time, the regulatory function of Treg cells is weakened, making the suppression of inflammation and autoantibody production less efficient. Abbreviations: CCR: C-C chemokine receptor; CXCL: C-X-C chemokine ligand; CXCR: C-X-C chemokine receptor; IFN: interferon; IL: interleukin; PD-1: programmed death-1; TNF: tumor necrosis factor; Tfh: follicular helper T cells; TGF-β: transforming growth factor-β; Th1: T helper 1 cells; Th2: T helper 2 cells; Th17: T helper 17 cells; Tph: peripheral helper T cells; Treg: regulatory T cells.

Dysregulation of various immune cell subsets plays a crucial role in the pathogenesis of SLE. In SLE, the subsets facilitating pro-inflammation or helping the proliferation and maturation of B cells are increased, while the regulatory subsets suppressing the excessive immune response are decreased (Figure 1) ^(9)^. The frequency, phenotype, and function of these cells reflect disease activity of SLE, suggesting that they could be potential biomarkers.

Plasmacytoid dendritic cells (pDCs) produce type I interferons (IFNs) in response to Toll-like receptor 7 (TLR7) and TLR9 stimulation. Type I IFNs activate pDCs, T cells, and B cells, and also play critical roles in the pathogenesis of SLE. A monoclonal antibody, anifrolumab, which blocks type I IFN signaling, was approved for treating SLE. Anifrolumab significantly reduced the disease activity of SLE ^(4)^.

T cells play important roles in the pathogenesis of SLE. T cells help to activate B cells, secrete inflammatory cytokines, and infiltrate organ tissues ^(1)^. T helper 17 (Th17) cells are a cluster of differentiation (CD)4^+^ T cell subset defined by their production of interleukin-17 (IL-17). An imbalance of Th17 and regulatory T (Treg) cells is also involved in the pathogenesis of SLE ^(10), (11)^. From a meta-analysis, patients with lupus nephritis or active SLE had an increased proportion of Th17 cells and a decreased proportion of Treg cells ^(10)^. Th17 cells have remarkable plasticity and can convert into multifunctional helper T cells or Th1 cells, which produce interferon γ (IFN-γ) ^(12)^. Pathogenic Th17 cells also produce IL-21, IL-22, and granulocyte-macrophage colony-stimulating factor. T helper 1 (Th1) cells are also involved in the pathogenesis of SLE. From a large-scale transcriptome analysis of 27 immune cell types from patients with SLE and healthy controls, Th1 cells are associated with disease activity, especially mucocutaneous and renal involvement ^(13)^. Interestingly, CD8^+^ T cells are also associated with disease activity ^(13)^. Double-negative T cells, CD3^+^ CD4^-^ CD8^-^ cells, are significantly increased in patients with SLE, and they produce IL-17. CD8^+^ T cells lose the expression of CD8, acquire a double-negative T cell phenotype, and infiltrate tissues ^(14)^.

Follicular helper T (Tfh) cells are characterized as CD4^+^ T cell subsets that facilitate the T-dependent B cell proliferation and maturation primarily by secreting IL-21. Human Tfh cells were first identified in the tonsil and express B cell lymphoma-6. In addition to C-X-C chemokine receptor 5 (CXCR5), programmed death-1 (PD-1), CD40 ligand (CD40L), inducible co-stimulator, and OX40 are also expressed on the surface of Tfh cells ^(15)^. Except for canonical Tfh cells localized in germinal centers, Tfh-like cells confirmed outside of germinal centers include extrafollicular Tfh cells and circulating Tfh (cTfh) cells. Despite various phenotypes and signature cytokines, cTfh cells share the same function as Tfh cells in the germinal centers in promoting differentiation of autoantibody-producing plasmablasts beyond the secondary lymphoid organs ^(16)^. The cTfh cells are functionally and phenotypically subdivided, according to the expression of CXCR3 and C-C chemokine receptor 6 (CCR6), specifically cTfh1 (CXCR3^+^CCR6^-^), cTfh2 (CXCR3^-^CCR6^-^), and cTfh17 (CXCR3^-^CCR6^+^) subsets ^(15)^.

While increased percentages of Tfh cells in correlation with B cell proportions and disease activity were detected in patients with SLE ^(17), (18)^, Bocharnikov et al. ^(19)^ declared that it was peripheral helper T (Tph) cells instead of Tfh cells that were correlated with disease activity status. The different molecular markers used to label target Tfh cells might explain this inconsistency to some extent. In addition to CXCR5 as an identifier of Tfh cells, the proportions of Tfh cells with expression of CCR7 ^(20)^, T cell immunoreceptor with Ig and immunoreceptor tyro-sine-based inhibitory domains (TIGIT) ^(21)^, Helios ^(22)^ (a member of the zinc finger transcription factor family) and NLRP3 ^(23)^ are also described to reflect immune status, indicating their potential roles as disease activity biomarkers.

With regard to cTfh cells, the significant expansion and activation of cTfh cells were observed in patients with active SLE, which altered after glucocorticoid treatment ^(24), (25)^. But more work is needed to unveil the incongruent results in various studies regarding one specific cTfh subset, especially the cTfh1 subset. Notably and interestingly, a clinical practice involving children and adults stressed that cTfh cell expansion was indicative of the disease activity in patients with active SLE, while in well-controlled SLE it showed no significance ^(26)^. Additionally, the imbalances between Tfh cells and follicular regulatory T (Tfr) cells ^(27)^ or Tregs ^(28)^ in SLE patients were elucidated in the form of ratios rather than a single lymphocyte subset, suggesting Tfh/Tfr and Tfh/Treg ratios as potential indicators and promising therapeutic targets.

Tph cells, initially discovered in inflamed rheumatoid arthritis joints, play a crucial role in lymphocyte recruitment and lymphoid follicle formation through CXCL13 production. The presence of CXCL13 is important for the activity of germinal centers, in which the CXCL13-CXCR5 chemokine axis plays a central role. Despite the similarities in functions with Tfh cells, Tph cells lack expression of Bcl-6 and CXCR5, and are phenotypically defined as PD-1^hi^CXCR5^-^CD4^+^ T cells. These cells contribute to pathological B cell activation and serve as potential biomarkers for T-B activation in autoimmune diseases, including SLE ^(29)^. The expansions of Tph populations, positively correlating with disease activity, were detected in patients with SLE according to several studies, and a correlation with renal damage severity reported in some studies ^(30), (31), (32), (33)^. Increased TIGIT ^(21)^-expressing and SLAM-associated protein (SAP) ^(34)^-expressing Tph cells and their therapeutic and predictive values were demonstrated, respectively. Longitudinal analyses have elucidated temporal fluctuations of Tph cells during disease flare, specifically showing relatively low levels before flares followed by significant increases during flares, indicating their potential in predicting the need for treatment and responses ^(35)^.

Recently, another interesting T cell subset was identified. Age-associated T helper (ThA) cells, a distinct CXCR3^mid^CD4^+^ effector memory T cell subset, were expanded with age ^(36)^. The patients with SLE had more ThA cells, and gene expression in ThA cells from these patients reflected disease activity ^(36)^.

Since one of the hallmarks of SLE is the production of a wide variety of autoantibodies, B cells are also requisite for the pathogenesis of SLE. In SLE, memory B cell populations are expanded, and atypical memory B cells, such as CD27^-^IgD^-^ (double-negative) B cells ^(37)^ and CD11c^+^ age-associated B cells, have a positive correlation with the disease activity ^(38), (39)^. Plasmablasts and plasma cells also play important roles in the pathogenesis of SLE. Importantly, B cell-targeted monoclonal antibodies and chimeric antigen receptor (CAR) T cells have efficacy in SLE, and the replenishment of the memory B cell population has been associated with disease relapse in SLE ^(39)^. In addition to anti-CD20 monoclonal antibody, rituximab, and B cell-activating factor (BAFF), belimumab, which can be used in Japan, several B cell-targeted therapies and CAR-T therapy are in ongoing clinical trials.

## Cytokines and Chemokines as Biomarkers in SLE

Cytokines and chemokines also play a pivotal role in the pathogenesis of SLE. They can be produced either by the innate immune system or the adaptive immune system, and function in immune cell differentiation and maturation, cytokine production, and signaling pathway induction. The abnormal profiles of cytokine and chemokine were emerging biomarkers to diagnose SLE and predict disease activity.

BAFF, also known as B lymphocyte stimulator, is a member of the tumor necrosis factor (TNF) family expressed on monocytes, macrophages, monocyte-derived dendritic cells, and neutrophils. Besides BAFF, the BAFF/APRIL system contains a proliferation-inducing ligand (APRIL), the BAFF receptor (BAFF-R), the transmembrane activator and calcium-modulating cyclophilin ligand interactor (TACI), and the B cell maturation antigen (BCMA). The interactions between factors and receptors trigger downstream signaling pathways and finally affect autoimmunity by promoting the survival, proliferation, and maturation of B cells ^(40)^. Several studies have reported the significantly elevated serum levels of BAFF, APRIL, and BCMA in patients with SLE. What is more, the increased cytokines showed an accordance with SLE disease activity index (SLEDAI) scores ^(41), (42), (43), (44)^. Taken together, the consistent results from these studies provided a relatively general conclusion that the serum levels of BAFF, APRIL, and BCMA had the potential to predict disease activity in patients with SLE. However, the direct evidence for TACI and BAFF-R being associated with disease activity is insufficient, and their clinical utilization remains unproven. Moreover, in patients with SLE, the aberrant expressions of BAFF and APRIL were reported to be associated with cardiovascular involvement, and the expression of BAFF solely was related to renal manifestations ^(41), (45)^. Notably, BAFF was investigated to be the most promising and powerful biomarker in the BAFF/APRIL system, not only in predicting disease activity but also in treatment response.

IFNs are a family of cytokines characterized by their defensive abilities against viral infection. So far, three main types of IFN systems have been identified, which are type I IFN system (including IFN-α, IFN-β, and IFN-Ω), type II IFN (namely IFN-γ), and type III IFNs (including IFN-λ1-4). The type I IFN system performs as a primary pathogenic factor in SLE, is frequently detected in the pre-disease state, and its abnormal presence is thought to be an alarm of predisposition to SLE initiation. Clinical data showed that both chronic and persistently elevated levels of type I IFNs and over-expression of type I IFN pathway genes were demonstrated in a great percentage of patients with SLE, further confirming its crucial role in the pathogenesis of SLE ^(46)^. Despite the initial susceptibility factor being verified already, whether type I IFNs have the ability to mirror the disease activity is still under research. Supplementary to previous cross-sectional studies declaring that type I IFNs were associated with disease activity but lacking longitudinal evidences, a recent research containing both cross-sectional and longitudinal studies has identified that type I IFNs, particularly IFN-α2 levels were significantly elevated in patients with SLE compared to healthy control, and their high levels were a promising predictor of an ongoing flares according to the longitudinal multivariable analysis ^(47)^. Another retrospective longitudinal study concerned with type I IFNs confirmed their associations with disease activity, but it argued that these correlations existed significantly only in treatment-naïve SLE patients, while the induction and maintenance of therapy made them less significant ^(48)^. Whittall Garcia et al. ^(49)^ have examined the performance of IFN-α in predicting lupus nephritis (LN) flares, and revealed that elevated serum IFN-α levels at the time of LN flares implied poor renal outcomes, including a higher frequency of developing LN flares. Another study with a cohort of 90 patients showed that IFN-α was performing better in predicting short-term in-hospital mortality than anti-dsDNA, complement components, and SLEDAI scores, highlighting its value in clinical pre-warning ^(50)^. However, the lack of efficient and standardized protocols rendered the serum IFN results confusing to interpret and complicated to harmonize with other studies. Hopefully, this problem can be addressed by improving techniques. In addition to type I IFNs, IFN-γ was also investigated for its diagnostic and predictive capacities in patients with SLE. According to a study centering on SLE patients, who were candidates for mesenchymal stem cells transplantation, the baseline IFN-γ levels may help to choose the suitable patients ^(51)^. To avoid the IFN detection problem, surrogate IFN-regulated markers have emerged as promising biomarkers for monitoring the disease activity, among which the interferon γ-inducible protein 10 (IP-10, also known as CXCL10), galectin-9 (Gal-9), and the sialic acid binding Ig-like lectin 1 (SIGLEC1) attracted much attention. Unfortunately, in a study conducted by Enocsson et al., ^(52)^ neither IP-10 nor Gal-9 showed a convincing association with disease activity. And instead of correlating with disease activity directly, the absence of SIGLEC1 was thought to be a promising and powerful tool to exclude SLE in suspected cases, with an area under the curve up to 0.95 ^(53)^.

The IL-1 family has similar functions to TLRs, primarily associated with innate immunity. So far there are 11 members of the IL-1 family of cytokines (IL-1 Receptor antagonist (IL-1Ra), IL-1α, IL-1β, IL-33, IL-18, IL-37, IL-36Ra, IL-36α, IL-36β, IL-36γ, IL-38), 10 members of the IL-1 family of receptors interacting with IL-1 family cytokines, and three decoy receptors (IL-1RII, soluble suppression of tumorigenicity 2 protein (sST2), IL-18BP) have been identified. The IL-1 family cytokines mainly function by targeting receptor-expressed cells, such as monocytes and lymphocytes, then provoking either anti-inflammatory or pro-inflammatory responses according to each cytokine’s preference ^(54)^. In patients with SLE, the serum levels of IL-1Ra, IL-18, IL-36α and IL-36γ were reported to be elevated in the active state and correlated with SLEDAI scores, indicating their potential as predictive biomarkers to monitor disease activity ^(55), (56), (57)^. Besides, IL-1Ra also showed an impressive performance in diagnosing SLE with a specificity of 92.9%, exceeding anti-dsDNA antibodies and C3 ^(58)^. The promising receptor candidates for predicting disease activity include soluble IL-1R (sIL-1R) 2 and sIL-1R4 ^(59)^. Soluble ST2 was also associated with disease activity; however, this correlation was confirmed only in pediatric SLE, and the clinical data with adult patients were not sufficient ^(60)^. But notably, IL-1 families were generally assumed to interpret disease status on a comprehensive scale but without any distinction between specific organ involvements, suggesting they might not be suitable for monitoring organ damage.

Besides the cytokines and chemokines discussed above, IL-6, IL-17, and IL-23 are available biomarker candidates as well. IL-6 involves itself in the immunity through mediating the activation and differentiation of lymphocytes. It has been reported that IL-6 was of great value in monitoring SLE disease activity, but without specific organ involvements ^(56)^. IL-17, produced mainly by Th17 subsets, functions by promoting inflammation and protecting T cells from apoptosis. A systemic review with meta-analysis verified that increased circulating IL-17 was associated with SLE disease activity but gave notice that the relationship was not absolute and further information was still in need ^(61)^. The IL-23/IL-17 axis is an emerging pathway and has been extensively studied in inflammatory and rheumatic diseases. The elevated levels of both IL-23 and IL-17 were confirmed in patients with SLE, and the results were described as LN-related ^(62)^.

## Autoantibodies as Biomarkers in SLE

Abnormal production and recognition of autoantibodies are one of the characteristic serological signatures associated with SLE. SLE-related antibodies, such as anti-dsDNA antibody and anti-Smith (anti-Sm) antibody, have been implemented as diagnostic criteria for decades. Nowadays, in addition to diagnosis, one important implication of recent results is that autoantibodies are promising clinical predictors associated with disease activity.

Since anti-dsDNA antibody was accepted in Systemic Lupus International Collaborating Clinics classification criteria version as an immunological signature in 2012, and successively adopted in European League Against Rheumatism/American College of Rheumatology (EULAR/ACR) classification criteria in 2019, the anti-dsDNA antibody has been utilized officially as a diagnostic and classification criteria for over 10 years ^(63), (64)^. However, whether anti-dsDNA antibody could authentically explain the pathogenesis of SLE and serve as a trustworthy diagnostic criterion for SLE is still under debate. The most enigmatic problem embedded in the anti-dsDNA antibody implementation for diagnosis is that anti-dsDNA antibodies are not unique to SLE. Bacterial and viral infections, and inflammatory diseases, were mentioned in the detection of anti-dsDNA antibodies. In addition to this, the heterogeneity of anti-dsDNA antibodies themselves extends the complexity, requiring standardized protocols and rational interpretations for laboratory tests. Turning now to the disease activity, recent studies have investigated anti-dsDNA over its fluctuations through the disease courses, particularly before and within the flares, and mentioned the predictive capability of anti-dsDNA in lupus nephritis management. Some opposite suggestions pointed out that the serum anti-dsDNA level was quite stable over the time course, regardless of disease activity and flares ^(65)^. But they also pointed out that coupling anti-dsDNA with other autoantibodies, such as anti-chromatin antibodies and anti-SSA/Ro60 antibodies, improved the predictive capacity of anti-dsDNA for disease activity and flares, suggesting that autoantibody profiles could be useful ^(65), (66)^.

Anti-Sm antibodies are autoantibodies against the Smith antigen, which was first established in the serum from a patient diagnosed with SLE in 1959 and was detected in approximately 5-30% of SLE patients according to recent studies. Like anti-dsDNA antibodies, anti-Sm antibodies were adopted as one of the immunological diagnostic standards in 2019 EULAR/ACR SLE classification criteria as well ^(64)^. Anti-Sm antibody is highly specific (>90%) to SLE but is accompanied by a low sensitivity. According to a longitudinal study, the anti-Sm antibody titer was correlated with baseline disease activity and subsequent alterations in disease status, which may help to assess the disease activity and improve the medication management ^(67)^. However, a clinical trial noted that in the belimumab treatment subgroup anti-Sm antibody showed no significance in correlating renal flares, compared to the placebo subgroup ^(68)^.

Other major autoantibodies involved in SLE include autoantibodies to small nuclear ribonucleoprotein (anti-snRNP), autoantibodies to ribosomal proteins (anti-RibP), and antiphospholipid antibodies (aPLs). Depending on the autoantigens, anti-snRNP are subdivided into anti-U1RNP, anti-U2RNP, and anti-U3RNP, among which the anti-U1RNP antibodies were especially associated with central neuropsychiatric manifestations ^(69)^. Anti-RibP are highly specific antibodies for diagnosing SLE but with a relatively low sensitivity. A well-documented meta-analysis demonstrated that anti-RibP was significantly associated with central nervous system (CNS) involvement ^(70)^. Both anti-U1RNP antibodies and anti-RibP antibodies demonstrated their potential in predicting neuropsychiatric status in patients with SLE in these studies. By binding to specific cell receptors, aPL mediate cell activation through several signaling transduction pathways. aPL is the defining feature of antiphospholipid syndrome but is also frequently detected in patients with SLE. A longitudinal study using machine learning identified a cluster with a high frequency of aPLs. This cluster had the highest frequency of anti-cardiolipin IgG/IgM, anti-β2-glycoprotein-1 (β2GPI) IgG/IgM, lupus anticoagulant, anti-phosphatidyl serine/prothrombin complex IgG/IgM, and anti-β2GPI-domain 1 IgG/IgM, and the highest frequency of seizures ^(71)^. Though the mechanism remains unclear, aPL is possibly associated with CNS manifestations.

## Complement Components as Biomarkers in SLE

**Figure 2.**
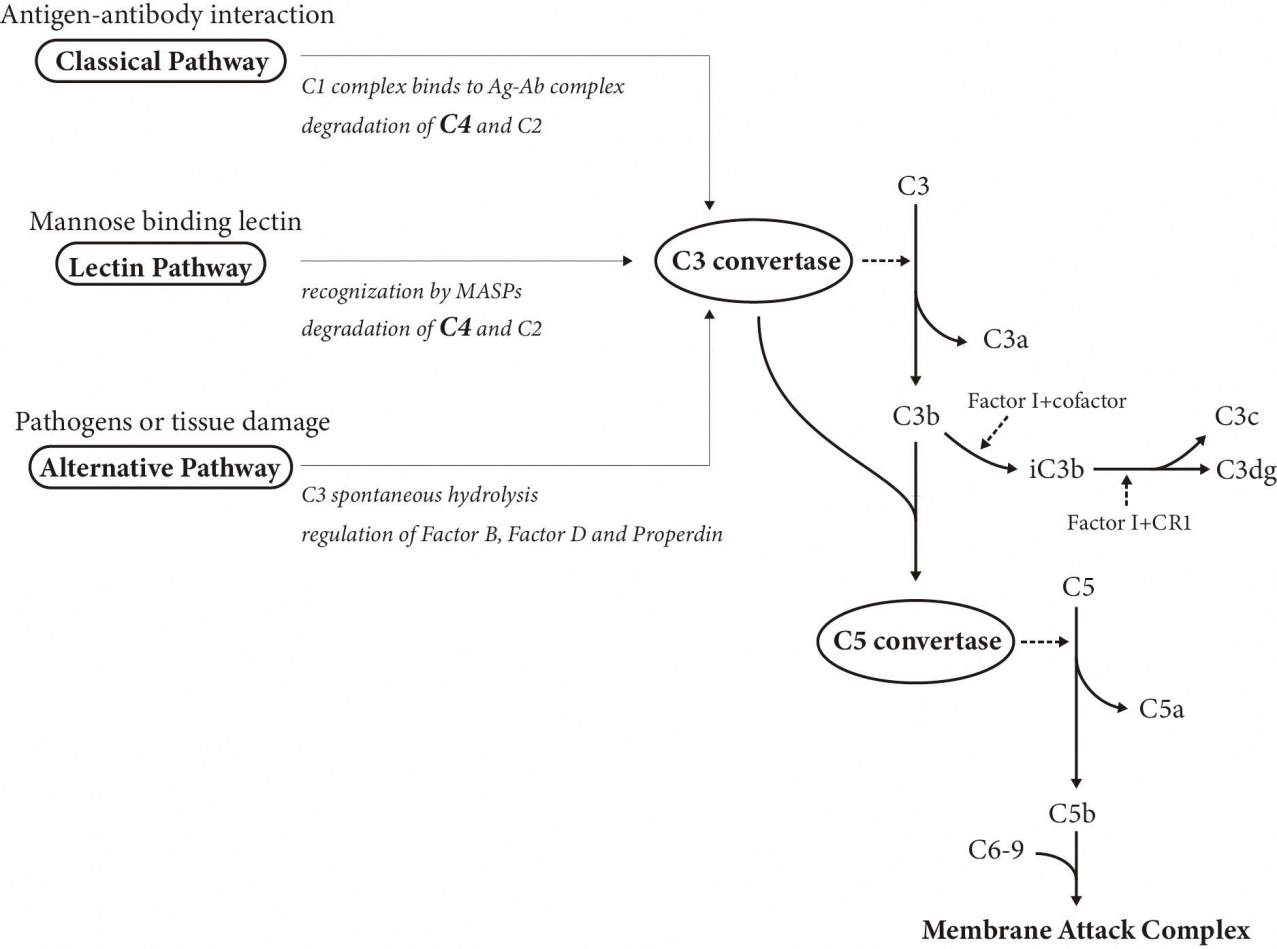
The complement cascade. The complement cascade can be initiated in three pathways, which are respectively described as the classical pathway, lectin pathway, and alternative pathway. The C3 convertase produced by each pathway promotes the degradation of C3. The C3b fragment sequentially constitutes the C5 convertase and participates in the following processes. The C3b fragment is quickly degraded to smaller fragments after its deposition, mediated by complement factor I and cofactors. Abbreviations: Ag: antigen; Ab: antibody; CR1: complement receptor 1; MASP: mannose-binding lectin-associated serine protease.

Complement system plays a crucial role in the pathogenesis of SLE by forming immune complexes (Figure 2), and its components have served as immunological criteria in diagnosing SLE and monitoring disease activity for years ^(64)^. Besides the traditional complement markers (i.e., C3, C4, CH50) that have already been accepted as biomarkers in practical use, novel biomarkers, including split products, have also emerged as promising and alternative predictors.

Due to the biphasic nature of complements, which means either activation or deficiency of complement components is likely to induce the development of SLE, interpretation of the complement tests requires a comprehensive understanding of the complement system. C3, C4, and CH50 are referred to as the traditional complement markers, and their abnormal decreases, hypocomplementemia, reflect disease activity. In contrast, novel biomarkers related to complement system contain complement components except C3 and C4 (e.g., C1q and C1s), complement split products (e.g., C3a and C3dg), and cell-bound complement activation products.

The degradation of C4 and C3 is of great importance in the complement cascade, facilitating the consequent formation of immune deposition. Low complement, generally defined as decreased C3, C4, or CH50 below the lower limit of normal for the testing laboratory, is an important assessment in diagnosing SLE and evaluating the disease activity status. However, it is noted that the CH50, C3, or C4 levels are not in accordance with disease status all the time, with frequent observations of normal complement levels in the active phase and hypocomplementemia in remission.

According to a recent study, the complement split product C3dg was superior to C3 as a diagnostic biomarker in differentiating SLE patients from healthy controls. Therefore, C3dg was proposed as a suitable alternative to conventional complement biomarkers in SLE diagnosis ^(72)^. The correlation between serum C3a levels and disease activity was confirmed by another study, which elucidated that C3a was able to differentiate active from inactive SLE and was valuable to monitor thrombophilia status ^(73)^.

## Conclusion

In this review, we discussed potential biomarkers in SLE, especially immune cell subsets, cytokines, chemokines, and complement. In actual clinical practice, it is very difficult to judge which treatment is best for each patient, which patients can have their glucocorticoid dose reduced, and which patients can achieve glucocorticoid-free remission. If we can use these potential biomarkers in the future, we can provide more efficient treatment strategies for patients with SLE.

## Article Information

This article is based on the study, which received the Medical Research Encouragement Prize of The Japan Medical Association in 2024.

### Conflicts of Interest

Michihito Kono reports grants and/or speaking fees from AbbVie Inc., Asahi-Kasei Co., Astellas Pharma Inc., AstraZeneca Plc., Ayumi Pharmaceutical Co., Ltd., Bristol-Myers Squibb Co., Ltd., Chugai Pharmaceutical Co., Ltd., Daiichi Sankyo Co., Ltd., Eisai Co. Ltd., Eli Lilly Japan K.K., Gilead Sciences K.K., GlaxoSmithKline K.K., Janssen Pharmaceutical K.K., Kowa Co. Ltd., Kyocera Co., Ltd., Lotte CO., LTD., Nippon Boehringer Ingelheim Co., Ltd., Nippon Shinyaku CO., LTD., Mitsubishi Tanabe Pharma Co., Mochida Pharmaceutical CO., LTD., Pfizer Inc., Sandoz., Taiju Life Social Welfare Foundation, Taisho Pharmaceutical, Takeda Pharmaceutical Co., Ltd., Terumo Co., Ltd., UCB Japan Co. Ltd. and Yamazaki Baking CO., LTD., outside the submitted work.

### Acknowledgement

This manuscript was developed with the support of artificial intelligence-assisted tools, such as Claude 3.7 Sonet (Anthropic, CA, USA), which facilitated grammar correction, refinement of expression, and overall improvement in the quality of the manuscript.

### Author Contributions

Yujie Song and Michihito Kono contributed equally to this work.

Michihito Kono conceived the research topic. Yujie Song and Michihito Kono contributed to the collection of related papers and manuscript preparation. All authors critically reviewed and revised the manuscript and approved the final version for submission.
